# Digital Twin Prospects in IoT-Based Human Movement Monitoring Model

**DOI:** 10.3390/s25216674

**Published:** 2025-11-01

**Authors:** Gulfeshan Parween, Adnan Al-Anbuky, Grant Mawston, Andrew Lowe

**Affiliations:** 1 Electrical and Electronics Engineering Department, Auckland University of Technology, Auckland 1010, New Zealand; gulfeshan.parween@autuni.ac.nz; 2 Physiotherapy Department, Auckland University of Technology, Auckland 0627, New Zealand; grant.mawston@aut.ac.nz; 3 Institute of Biomedical Technologies, Auckland University of Technology, Auckland 1010, New Zealand; andrew.lowe@aut.ac.nz

**Keywords:** wearable sensors, artificial intelligence, digital twin, IoT, human movement monitoring, prehabilitation

## Abstract

Prehabilitation programs for abdominal pre-operative patients are increasingly recognized for improving surgical outcomes, reducing post-operative complications, and enhancing recovery. Internet of Things (IoT)-enabled human movement monitoring systems offer promising support in mixed-mode settings that combine clinical supervision with home-based independence. These systems enhance accessibility, reduce pressure on healthcare infrastructure, and address geographical isolation. However, current implementations often lack personalized movement analysis, adaptive intervention mechanisms, and real-time clinical integration, frequently requiring manual oversight and limiting functional outcomes. This review-based paper proposes a conceptual framework informed by the existing literature, integrating Digital Twin (DT) technology, and machine learning/Artificial Intelligence (ML/AI) to enhance IoT-based mixed-mode prehabilitation programs. The framework employs inertial sensors embedded in wearable devices and smartphones to continuously collect movement data during prehabilitation exercises for pre-operative patients. These data are processed at the edge or in the cloud. Advanced ML/AI algorithms classify activity types and intensities with high precision, overcoming limitations of traditional Fast Fourier Transform (FFT)-based recognition methods, such as frequency overlap and amplitude distortion. The Digital Twin continuously monitors IoT behavior and provides timely interventions to fine-tune personalized patient monitoring. It simulates patient-specific movement profiles and supports dynamic, automated adjustments based on real-time analysis. This facilitates adaptive interventions and fosters bidirectional communication between patients and clinicians, enabling dynamic and remote supervision. By combining IoT, Digital Twin, and ML/AI technologies, the proposed framework offers a novel, scalable approach to personalized pre-operative care, addressing current limitations and enhancing outcomes.

## 1. Introduction

Abdominal cancer is among the most prevalent and life-threatening cancers worldwide, disproportionately affecting middle-aged and older adults and remaining a leading cause of mortality [1,2,3,4,5]. Surgery is the cornerstone of curative treatment for locally advanced cases [3,6], yet post-operative complications affect 30–50% of patients, particularly those with reduced cardiorespiratory fitness [6]. Pre-operative therapies such as chemotherapy can further impair physical function, highlighting the need for interventions that optimize capacity before surgery [7,8].

Prehabilitation refers to a set of structured pre-operative interventions designed to enhance patients’ physiological resilience and optimize functional capacity prior to surgery. It has been shown to significantly improve surgical outcomes, reduce post-operative complications, shorten hospital stays, and enhance overall quality of life. Evidence from supervised clinical programs demonstrates measurable improvements in functional performance and reductions in adverse surgical outcomes [3,4,6,7,9,10,11,12,13]. For example, a pilot study by Carli et al. (2020) [4] reported a substantial decrease in post-operative complications from 70.2% to 37.5% among patients undergoing complex colorectal procedures. Prehabilitation typically incorporates aerobic and resistance training modalities, delivered either in supervised clinical environments or through home-based programs. However, both models present inherent limitations. Supervised programs, while clinically effective, are often constrained by geographic isolation, limited healthcare resources, and logistical barriers. Conversely, unsupervised home-based interventions tend to suffer from reduced patient adherence and lack of real-time monitoring, which may compromise their efficacy [5,10].

Recent advancements in digital health technologies, particularly wearable devices, the Internet of Things, and wireless sensor networks, have enabled the emergence of technology-integrated prehabilitation contributing mixed-mode models that address limitations inherent in both supervised and unsupervised approaches [8,14,15,16,17,18,19]. Specifically, mixed-mode IoT-based prehabilitation programs combine remote monitoring and clinician supervision throughout the complete session, thereby bridging gaps in accessibility and adherence [20,21]. These systems reduce the need for frequent in-person visits, alleviate transportation burdens, and optimize resource utilization within healthcare settings. Wearable sensors capture patient activity data, which is processed locally via edge or gateway devices and analyzed in the cloud to generate actionable insights. This architecture supports personalized exercise regimens, enhances patient engagement, and facilitates continuous supervision. Early studies have demonstrated that wearable-enabled prehabilitation can lead to functional improvements in patients undergoing abdominal cancer surgery [17,18,19,22,23]. Moreover, recent innovations in wearable technology have provided oncologists with real-time, objective data on patient activity and exercise intensity [18]. Some studies have also explored the financial feasibility of teleprehabilitation, particularly for high-risk colorectal cancer patients, suggesting its potential cost-effectiveness. However, challenges remain in ensuring adherence to prescribed exercise intensity and duration [16]. While preliminary research has shown that IoT devices can effectively capture and interpret movement data in both abdominal cancer patient’s prehabilitation and post-fracture rehabilitation contexts [20,21,22,23], current implementations remain limited in scope and adaptability. Despite these promising developments, existing models often rely on static protocols and lack mechanisms for real-time adjustment and personalization. In addition, few studies have explored the integration of advanced AI/ML techniques for precise activity recognition and personalized intervention planning, highlighting a critical gap that this study seeks to address.

Within this evolving technological landscape, the concept of the Digital Twin has emerged as a transformative innovation with significant potential for healthcare applications. Originally developed in industrial domains, DT refers to dynamic, virtual representations of physical systems that are continuously updated with real-time data from their physical counterparts. In some implementations, DTs also exert bidirectional feedback, enabling interaction and influence between virtual and physical systems [24,25,26,27,28]. In healthcare, DT integration is increasingly conceptualized as part of a flexible, intelligent, and patient-centered ecosystem that connects individuals with clinical teams through real-time data exchange and simulation capabilities [27]. A specific adaptation of this concept, Human Digital Twins (HDTs), enables the creation of evolving, individualized models that support early detection, predictive simulation, and personalized intervention planning [29,30,31]. Although still in its early stages, DT applications have shown promise across various domains, including cloud-based elderly care systems [32], electrocardiogram (ECG) monitoring [27,33], vulnerability detection in lung cancer [34], and fitness management [29]. Furthermore, DT-enabled frameworks have been proposed for rehabilitation robotics, such as self-balancing exoskeletons that enhance patient–robot interaction [31] and conceptual models for prehabilitation support before surgery [35]. DTs have also been applied in IoT-enabled environments, such as smart campuses, where virtual models of connected devices improve energy efficiency and operational management [36]. When combined with simulation and AI, DTs offer enhanced capabilities for data interpretation, explainable AI, and handling sparse or missing data [37]. Taken together, these developments suggest that DT technology, particularly when integrated with IoT and AI, holds substantial promise in advancing personalized, adaptive, and real-time management of prehabilitation systems.

To address the limitations of current mixed-mode IoT-based prehabilitation models, this study proposes a novel conceptual framework that integrates Digital Twin and Artificial Intelligence technologies atop IoT-based monitoring systems for prehabilitation of abdominal cancer patients. Unlike existing approaches that rely on static, generalized protocols, the proposed framework enables the following:

- Dynamic, personalized, and data-driven model, continuously aligned with evolving patient movement data.
- Automated feedback and precise activity recognition using advanced machine learning algorithms, overcoming limitations of traditional FFT-based methods.
- Individualized intervention planning through real-time simulation and bidirectional communication between patients and clinicians.
- Scalable and remote care, bridging the gap between monitoring and intelligent decision support.

This framework introduces a new paradigm in prehabilitation by combining IoT, DT, and ML/AI technologies to provide adaptive, personalized, and real-time management of pre-operative care, specifically tailored to the needs of prehabilitation of patients with abdominal cancer.

## 2. Related Work

### 2.1. Prehabilitation Program Structure: Clinical and Technological Needs

Pre-rehabilitation programs aim to improve the physical fitness of patients prior to surgery, with aerobic capacity being a key predictor of surgical resilience and post-operative outcomes. Although structured exercise programs are increasingly adopted in clinical settings, especially for frail patients, the evidence supporting specific modalities and delivery methods remains inconsistent [3,4,7,11]. Aerobic exercises such as treadmill walking, cycling, and rowing are commonly prescribed, but few studies justify the selection of modality or compare their effectiveness. Interval training is gaining popularity, but its benefits over continuous training in surgical populations are underexplored. Resistance training is often included to improve muscular strength, typically at moderate intensity (10–20 repetitions) [11,12,38]. However, its impact varies between studies and the interaction between aerobic and resistance modalities is not well understood due to the lack of standardized protocols.

Supervised programs in clinical or prehabilitation settings offer professional oversight and standardized assessments like Cardiopulmonary Exercise Testing (CPET) and 6 Minute Walk Test (6MWT), resulting in higher adherence and improved outcomes [3,4,12]. However, they are resource-intensive and often inaccessible to patients in remote areas. Socioeconomic and cultural factors influencing participation are often overlooked, limiting generalizability. On the other hand, home-based programs offer flexibility and overcome geographic barriers, but often suffer from poor adherence and limited supervision [5,14,17]. The assumption that patients can self monitor effectively is questionable, and the role of digital tools, such as wearables, mobile apps, and telehealth platforms, in enhancing engagement is underexplored. As summarized in Table 1, supervised programs consistently show improvements in aerobic capacity and reductions in complications, while unsupervised and technology-based models yield mixed results. This highlights the need for hybrid models that combine the accessibility of home-based care with the clinical rigor of supervised interventions.

Technological advances, particularly in remote monitoring, offer promising solutions. Wearable sensors, mHealth apps, and telemedicine platforms enable real-time tracking of movement, physiological metrics, and exercise compliance [8,14,15,16,17,18,19,39]. For example, the Digital Platform for Exercise (DPEx) demonstrated the feasibility of decentralized, patient-centric delivery of exercise therapy across cancer cohorts [15]. A bimodal tele-prehabilitation program for high-risk colorectal cancer patients achieved high adherence and satisfaction [16]. Systematic reviews and pilot studies show that wearable activity monitors can improve physical activity and predict clinical outcomes, though standardization issues persist [17,18,19].

IoT-enabled systems further reduce barriers by supporting continuous monitoring and mixed-mode prehabilitation delivery, especially for abdominal cancer patients with limited access to healthcare resources [20]. Monitoring of prehabilitation performance is increasingly supported by mathematical models that quantify effort and provide real-time feedback on exercise type and intensity, as illustrated in Table 2 [38]. Table 2 also highlights the diversity of exercise modalities such as walking, cycling, rowing, and resistance training and the flexibility of delivery locations, including clinics, gyms, parks, and home environments. A credit-based performance measurement system, though not yet standardized, shows promise for quantifying patient effort and progress. When integrated with remote monitoring technologies, this system supports personalized feedback and adaptive programming. In addition, IoT-enabled environments, including those used in hip fracture rehabilitation, demonstrate the feasibility of continuous movement tracking across both supervised and unsupervised settings. These systems offer flexible analysis, visualization, and feedback mechanisms that enhance adherence and recovery outcomes [21].

These structured components form the foundation of mixed-mode prehabilitation models, ensuring consistency while allowing for customization. When combined with IoT platforms and AI-driven Digital Twin frameworks, they enable individualized prehabilitation scenarios, predicting outcomes, and delivering adaptive feedback in real time. This integration of IoT, AI, and DT technologies supports personalized, remotely monitored interventions and provides actionable insights to optimize patient outcomes in mixed-mode prehabilitation settings [39].

### 2.2. IoT-Based Prehabilitation Model Architecture

The Internet of Things has emerged as a transformative force in healthcare, enabling continuous patient monitoring, real-time data acquisition, and cloud-based management across clinical and home environments [40]. By leveraging wearable sensors, edge computing, and cloud analytics, IoT supports scalable remote care particularly valuable in prehabilitation settings. Despite its potential, several challenges hinder widespread adoption. Surveys have identified a lack of standardization in wearable devices, concerns regarding data security and privacy, and usability issues that impact patient engagement [41]. A comprehensive review of IoT- and IoMT-based smart healthcare systems has outlined key system architectures, data fusion techniques, and future directions for intelligent, connected care [42].

Advanced IoT frameworks have demonstrated promise in prehabilitation. Publish–subscribe communication models facilitate efficient multi-user tracking by transmitting only relevant data, thereby reducing network congestion [43]. The Digital Human Model (DHM) integrates motion capture, IoT devices, machine learning, and virtual reality to simulate and optimize human movement [44]. In post-operative hip fracture care, IoT-enabled systems combine wearable sensors and edge computing to deliver personalized feedback and real-time supervision [21].

These developments underscore the potential of IoT in mixed-mode prehabilitation for abdominal cancer patients. Systems integrating wearable sensors and cloud platforms have shown improved adherence and functional outcomes during six-week pre-operative programs [20]. However, current implementations often rely on static protocols and lack personalization, dynamic modeling, and integration with clinical decision-making, highlighting a critical research gap.

To address these limitations, the integration of potential functionalities of Digital Twin technology is proposed. DTs enable real-time simulation of patient-specific prehabilitation scenarios, predictive analytics, and adaptive feedback. When combined with IoT and machine learning, DTs can transform passive monitoring systems into intelligent, patient-centered platforms enhancing adherence, optimizing outcomes, and expanding access to personalized pre-operative care [39].

**Figure 1:**
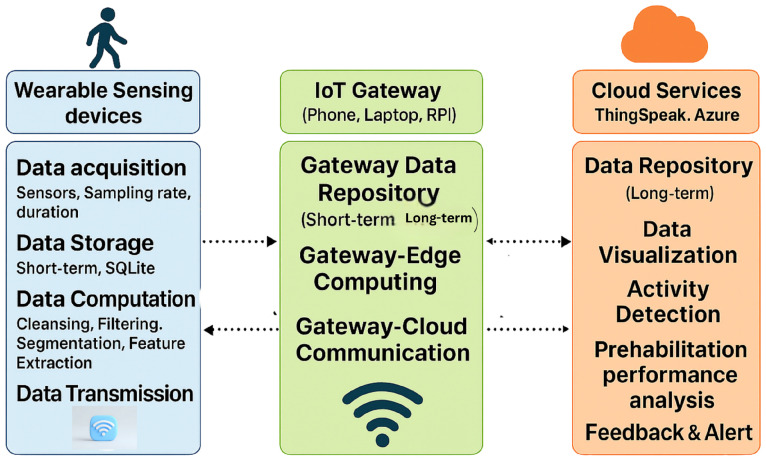
IoT architecture: The system integrates wearable sensors, IoT gateways, and cloud services to enable real-time data acquisition, processing, and feedback for patient activity and performance tracking.

The foundational architecture of an IoT-enabled prehabilitation system typically follows a layered sensor–edge–cloud model as shown in Figure 1, supporting continuous monitoring across clinical, residential, and home environments [20,21].

- Sensing Layer: The sensing layer comprises wearable devices such as inertial sensors, which are responsible for capturing physiological and movement-related data in real time. This layer may also support short-term data storage and basic preprocessing functions, such as noise filtering and signal normalization. The effective implementation of this layer depends on several critical factors, including appropriate sensor selection, optimal placement on the body, accurate sampling rates, and reliable data storage mechanisms. These considerations are essential to ensure data fidelity, minimize artifacts, and support downstream analytics in prehabilitation and remote monitoring systems.
- Edge/ IoT Gateway: This layer serves as the intermediary between the sensor network and the cloud infrastructure. Sensors interface with edge nodes such as microcontrollers or Raspberry Pi devices to perform localized computation, noise filtering, and feature extraction FFT. Additionally, preliminary inference and data aggregation are conducted at this stage to reduce communication latency and minimize the volume of data transmitted to the cloud, thereby enhancing system efficiency and responsiveness.
- Cloud Layer: Preprocessed data from edge or sensors are securely transmitted to cloud platforms for long-term storage, advanced analytics, and visualization. In prehabilitation systems, this layer plays a critical role in enabling real-time analysis of patient performance, supporting feedback loops, and facilitating clinician oversight. Cloud-based infrastructures allow for scalable data processing, can integrate with machine learning models, and allow for the deployment of personalized rehabilitation protocols. Additionally, performance dashboards and interactive interfaces provide both clinicians and patients with actionable insights, enhancing adherence and optimizing therapeutic outcomes. Ensuring data security, interoperability, and low latency access remains essential for effective implementation in mixed-mode settings.

### 2.3. Human Movement Recognition: Role of ML/AI

Accurate human movement recognition (HMR) is essential for IoT-enabled prehabilitation systems, as it enables real-time monitoring and supports the development of personalized prehabilitation models. Advances in wearable sensors such as accelerometers, gyroscopes, and electromyography combined with an Intelligent Algorithm (ML/AI) have significantly enhanced activity classification capabilities within HMR systems. Recent studies have systematically reviewed the methods, trends, and applications in this domain [45,46,47,48,49,50].

Numerous studies have demonstrated the effectiveness of ML and AI models in recognizing activities such as walking, sitting, and stair navigation. For example, Sukor et al. (2018) [46] achieved 90% accuracy using graph-based and statistical classifiers with smartphone accelerometer data. Similarly, Khan et al. (2022) [47] compared convolutional neural networks (CNNs), artificial neural networks (ANNs), deep neural networks (DNNs), and deep belief networks (DBNs), identifying DNNs as the most effective, with an accuracy of 96%. Furthermore, 96.4% accuracy was achieved using CNNs and emphasized the importance of edge computing in enhancing real-time performance [51]. Beyond accuracy, fairness in activity recognition particularly for older adults with varying functional abilities is an emerging concern. Alam et al. (2020) [50] proposed an AI fairness framework using Bi-LSTM models to detect multi-label activities with a single wearable sensor, demonstrating improved recognition across diverse populations.

Table 3 summarizes key studies on sensor-based activity recognition, highlighting the technologies, methods, and performance metrics employed. These approaches form the intelligent layer of IoT-based prehabilitation systems, enabling adaptive, data-driven interventions tailored to individual patient needs.

### 2.4. Digital Twins in Smart Healthcare and Prehabilitation

**Figure 2:**
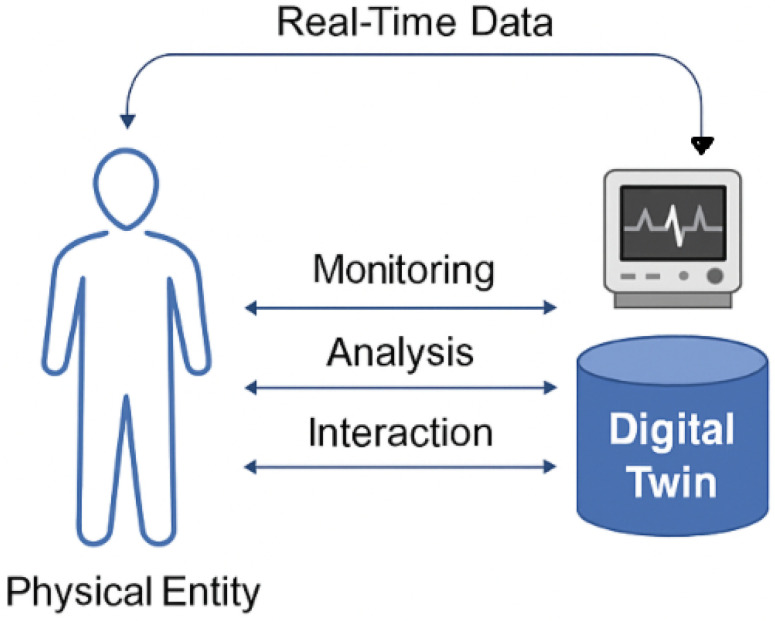
The general framework of Digital Twin technology. Real-time data from a physical entity (e.g., a patient) is continuously captured and transmitted to its digital counterpart. The Digital Twin enables ongoing monitoring, analytical processing, and interactive intervention and feedback, forming a closed-loop system that supports personalized and adaptive care.

Digital Twin technology has emerged as a transformative innovation across multiple domains, with particularly impactful applications in healthcare and prehabilitation. Qi et al. (2022) [25] provide a foundational overview of DT systems, detailing their architecture, communication protocols, and cross-domain applicability. Other authors have focused on the conceptual development, enabling technologies, and industrial applications of DTs [26]. Figure 2 illustrates the core concept of Digital Twin technology in healthcare. Real-time data from a physical entity such as a patient is continuously captured and transmitted to a digital counterpart. This Digital Twin enables ongoing monitoring, analytical processing, and interactive feedback, forming a closed-loop system that supports personalized and adaptive care.

In healthcare, DTs are increasingly employed to construct adaptive digital representations of patients and medical devices, facilitating real-time monitoring, enhanced diagnostics, and personalized treatment planning [27,34,52]. For example, Liu et al. (2019) [32] introduced the CloudDTH framework, which integrates wearable IoT devices to enable continuous health monitoring in elderly care. This system dynamically updates patient profiles and supports predictive clinical interventions. Similarly, the SmartFit platform utilizes DTs to monitor athletes’ physical and behavioral data through IoT sensors and user-input applications. Although originally designed for sports optimization, its architecture demonstrates strong potential for broader applications in health monitoring and rehabilitation contexts [29].

Further research explores DTs in rehabilitation, including a virtual supervision model for exoskeleton-assisted therapy. This approach supports remote kinesiotherapy and incorporates safety mechanisms to prevent adverse events, highlighting DTs’ role in remote and adaptive care [31]. DTs are increasingly integrated with IoT systems to enhance operational efficiency. Conceptual models facilitate the synchronization of heterogeneous data sources, enabling predictive analytics and fault detection [27,28,35,53]. In educational settings, DTs have been proposed to automate IoT devices for infrastructure management, such as lecture halls, offering improved visibility and control [36]. Moreover, AI-powered DT frameworks for smart homes demonstrate high accuracy in detecting irregular ECG rhythms, showcasing the potential of DTs in health-focused smart environments [33]. Table 4 summarizes key studies that illustrate the breadth of DT applications across healthcare and prehabilitation.The table categorizes each contribution by domain, integration type, technologies employed, and outcomes achieved. These examples collectively highlight the transformative potential of DTs in prehabilitation and healthcare, enabling predictive analytics, personalized interventions, and enhanced system responsiveness. The integration of DTs with IoT and AI technologies not only improves clinical decision-making but also supports scalable, remote, and patient-centric care models.

## 3. IoT Framework for Adaptive Prehabilitation Interventions Using Digital Twin

### 3.1. Conceptual Framework

**Figure 3:**
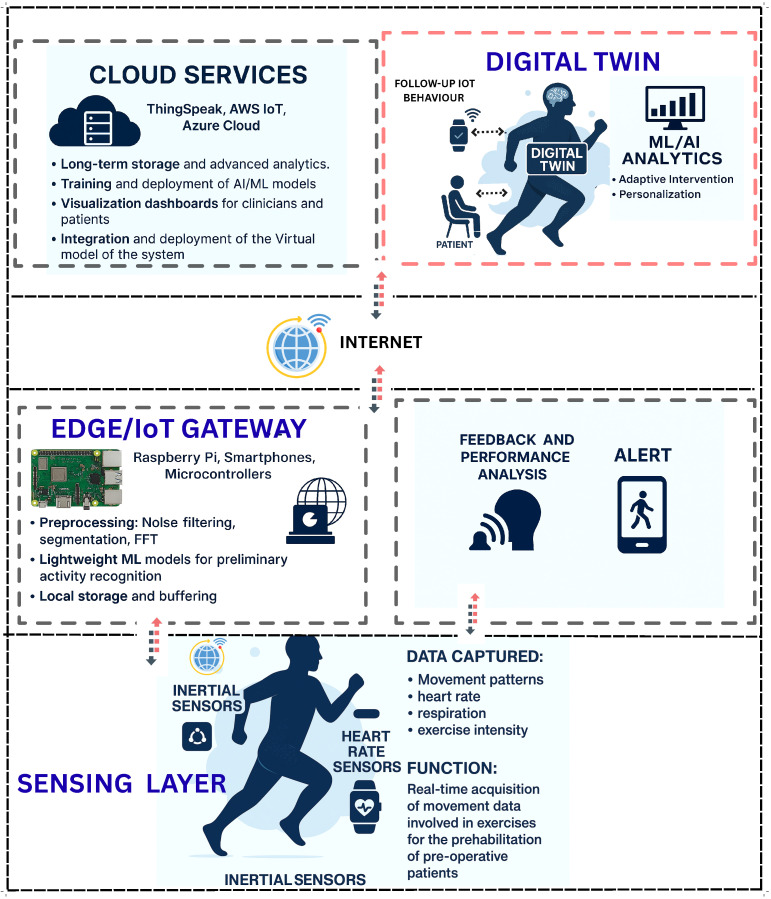
The figure illustrates a multi-layered architecture integrating cloud services, Digital Twin technology, decision support systems, edge/IoT gateways, sensing devices, and patient engagement tools.

This section presents a multi-layered conceptual framework for IoT-enabled prehabilitation systems augmented by Digital Twin (DT) technology. The framework spans data acquisition, communication, edge/cloud processing, DT modeling, analytics and decision support, and clinical application, as illustrated in Figure 3. It is designed to operate in mixed-mode settings, enabling continuous monitoring, accurate human movement recognition, and personalized interventions [20,21,35].

#### 3.1.1. Sensing Layer

The sensing layer constitutes the foundational component of the proposed framework, responsible for capturing multimodal data related to human movement and physiological parameters. Ubiquitous mobile and wearable devices, such as smartphones and smartwatches, serve as primary data acquisition tools, equipped with embedded sensors, including accelerometers, gyroscopes, magnetometers, GPS modules, and heart rate monitors [48,49]. Among these, accelerometers have demonstrated high classification performance in human movement recognition tasks, achieving accuracies of approximately 92%. When combined with gyroscopic data through multimodal sensor fusion, the classification accuracy can further improve to around 95% [46]. In the context of prehabilitation, common physical activities include walking, treadmill exercises, cycling, rowing, and resistance training, which are generally performed at varying intensities (see Table 2) [20,21,35,38]. During these activities, raw sensor signals are continuously sampled over program durations ranging from four to six weeks. The resulting data streams include triaxial linear acceleration (X, Y, Z), angular velocity, heart rate, and geolocation coordinates. These signals are primarily sourced from wearable sensors and form the basis for both movement classification and physiological monitoring. To ensure efficient data handling and compatibility with downstream processing pipelines, raw sensor data is stored locally on the participant’s device using structured formats such as CSV (for broad compatibility), SQLite (for efficient querying of large datasets), and JSON (for lightweight data exchange in IoT environments). On-device preprocessing is employed to reduce computational overhead and preserve device battery life and bandwidth. This includes noise filtering (e.g., low-pass filtering to remove high-frequency artifacts), DC offset removal (to correct baseline drift), segmentation (partitioning continuous data streams into fixed or adaptive windows), and feature extraction (deriving statistical, temporal, and frequency domain features for machine learning and AI-based inference). The primary sensor data types include the following.

- Accelerometer readings: Three-axis values (X, Y, Z) indicating linear acceleration.
- Gyroscope data: Angular velocity for detecting rotational movements.
- Heart rate: Captured heart rate sensors for physiological context.

To validate the design and functionality of the sensing layer, preliminary experiments were conducted using wearable sensors to collect motion data during a range of physical activities, as discussed in Section 4 and Section 5. The resulting dataset comprises three primary components. First, raw data were continuously acquired from triaxial accelerometer signals sampled at 20 Hz, capturing acceleration along the X, Y, and Z axes. Second, the data were segmented into windowed intervals ranging from 3 to 10 s to facilitate structured analysis and feature extraction using FFT. Third, frequency domain characteristics were derived from each segment using Fast Fourier Transform, enabling the identification of dominant frequency components associated with different movement patterns and recognition of activity based on the dominant frequency associated with movement patterns. These initial results demonstrate the feasibility of the sensing layer in capturing high-quality motion data and support its integration into the broader Digital Twin framework for real-time monitoring and adaptive feedback in prehabilitation contexts.

#### 3.1.2. IoT Gateway or Edge Level

In the proposed framework, the edge or gateway layer bridges wearable devices and cloud services, enabling low-latency processing and real-time responsiveness. Wearable sensors (e.g., accelerometers, gyroscopes) transmit raw motion data to edge nodes (e.g., Raspberry Pi), where lightweight preprocessing is performed, including noise filtering, segmentation, and FFT-based feature extraction to reduce transmission overhead and preserve data fidelity.

To support real-time, personalized monitoring, the framework incorporates edge-based activity recognition using ML/AI models deployed directly on gateway devices. A CNN model achieves 96.4% accuracy in classifying static and dynamic activities, with 8-bit quantization reducing model size and enabling efficient on-device inference [51]. This minimizes reliance on cloud resources, enhances privacy, and enables adaptive feedback within the Digital Twin-enabled system.

Traditional FFT-based methods yield approximately 78% accuracy but are limited by signal overlap and manual feature engineering [20,21]. To overcome these constraints, the framework integrates ML models capable of learning patterns from both time and frequency domain data, including raw sensor inputs. These models are trained and validated prior to deployment and integrated into the Digital Twin for continuous monitoring and feedback. A range of algorithms, including SVM, Random Forests, CNNs, and LSTMs, can be employed to ensure robust activity classification.

#### 3.1.3. Cloud-Level and Digital Twin Interaction

The cloud layer plays a pivotal role in enabling intelligent interaction between IoT devices and advanced analytics through the Digital Twin. Acting as a central intelligence hub, the DT continuously receives real-time data from wearable sensors, such as accelerometers and heart rate monitors. This data is used to construct and update a dynamic virtual model of the physical system. Simultaneously, ML/AI algorithms validate and refine the incoming data by filtering noise, detecting anomalies, and classifying movement patterns to ensure accurate representation and analysis.

The proposed framework aggregates sensor data from wearable devices to provide scalable storage, advanced analytics, remote monitoring capabilities, and performance evaluation at the cloud level. While edge devices perform real-time activity recognition, the cloud supports computationally intensive ML models for enhanced recognition accuracy, personalized modeling, performance analysis, and model training and deployment. The cloud also enables continuous performance tracking, detects deviations in behavioral patterns, and supports personalized program models for scheduled prehabilitation monitoring. Additionally, it facilitates user interaction through a feedback mechanism. This framework encompasses general cloud functionalities while extending them through the integration of Digital Twin technology.

The implementation of the Digital Twin at the cloud level enables continuous monitoring and follow-up of IoT device behavior, allowing for dynamic system management and auto-intervention when necessary. The DT processes incoming data using ML algorithms and advanced analytics. It can train on patient-specific data to personalize the virtual model. Upon identifying deviations, the DT initiates automated feedback mechanisms, sending adaptive instructions back to the wearable device or gateway, such as modifying exercise intensity or issuing alerts. This closed-loop interaction ensures that the DT not only visualizes and monitors patient performance but also actively manages and personalizes prehabilitation in real time, thereby enhancing clinical decision-making and patient outcomes.

For this study, ThingSpeak (TS), an IoT cloud platform, is employed to collect and store real-time data while facilitating data processing and visualization. MATLAB R2024b is incorporated into the system for computational analysis and model training. TS supports up to eight data fields, three location fields, and one status field, and operates via communication channels. It efficiently reduces latency in transmitting processed data from the gateway to the cloud by updating data every second and handling approximately 90,000 messages daily [20,21].

For initial prototype testing, a Simulink MATLAB App has been developed to transmit raw accelerometer data to the cloud through a Simulink model detailed in Section 6. The collected data is stored in the ThingSpeak cloud for visualization and further analysis. By applying advanced ML algorithms and adaptive learning techniques, the digital model can be updated according to the physical system’s requirements and provide feedback on prehabilitation performance. This operational Digital Twin offers a dynamic and comprehensive virtual representation of the system.

The integration of cloud intelligence with the Digital Twin ensures not only accurate recognition and visualization of patient activities but also dynamic management and automated intervention by allowing the follow-up of the IoT system. This synergy personalizes prehabilitation, reduces clinical risks, and improves overall treatment outcomes.

## 4. Data Acquisition Method

Effective data collection is foundational to the operation of an IoT-based system interacting a DT, particularly in healthcare applications such as prehabilitation. The process begins at the sensing level, where wearable IoT devices, such as accelerometers and physiological sensors, continuously capture real-time data related to patient movements and heart rate. This raw data is transmitted to edge/cloud via IoT communication platforms, which serve as intermediaries for secure and scalable data transfer.

Once in the cloud, data is integrated to construct and update a dynamic virtual representation of the physical system. This integration enables the DT to mirror the physical system, facilitating the continuous monitoring and follow-up of prehabilitation progress. The DT not only visualizes the data but also interprets it in context, dynamically managing the complete physical IoT system and allowing auto-intervention. The model is further tested with raw data integration and personalized.

Simultaneously, the collected data undergoes advanced processing using ML/AI algorithms. These computational models validate the integrity of incoming data by filtering out noise, detecting anomalies, and classifying activity patterns. The validated data can be further used to refine the DT model, enabling adaptive learning and personalized feedback. This dual pathway of data integration into the DT and ML/AI-based validation ensures that the system remains responsive, intelligent, and capable of delivering targeted interventions in real time.

**Figure 4:**
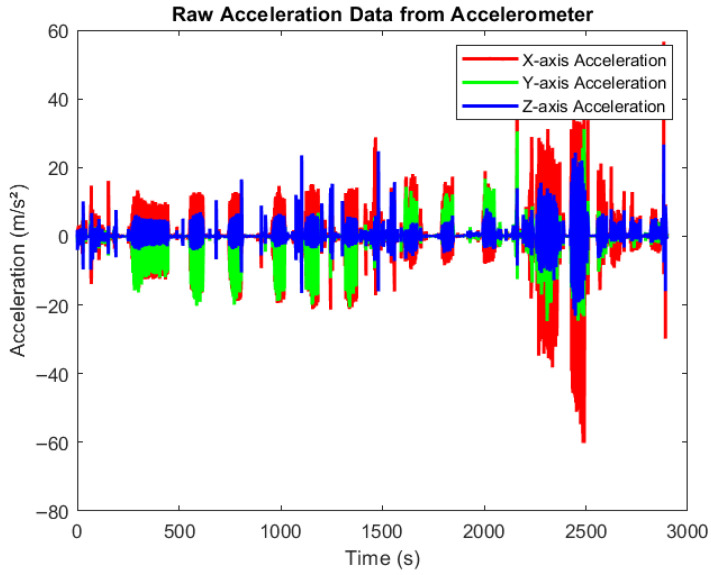
Raw data from accelerometer. Time-series plot showing raw acceleration data collected from an accelerometer across three axes: X (red), Y (green), and Z (blue). The x-axis represents time (0–3000 s), and the y-axis shows acceleration values in meters per second squared (m/s^2^). The signal exhibits dynamic fluctuations and spikes, reflecting different movements pattern in different activities.

Figure 4 presents raw accelerometer data collected during various physical activities using smartphones equipped with the MATLAB Mobile application. The devices were securely positioned to capture motion across three axes, X, Y, and Z, in real time. Participants engaged in activities such as walking, cycling, and rowing at varying intensity levels (low, medium, and high). The figure clearly illustrates how acceleration values fluctuate with activity intensity. Very low acceleration corresponds to resting states, moderate variations indicate medium-paced movements, and higher spikes reflect high-intensity exercises. All data were automatically synchronized to MATLAB Drive for offline processing and further analysis.

## 5. Data Analysis and Processing

Data processing is critical for transforming raw sensor inputs into meaningful insights. In this proposed framework, two complementary approaches will be employed: traditional signal processing using Fast Fourier Transform, and advanced machine learning/Artificial Intelligence techniques.

**Figure 5:**
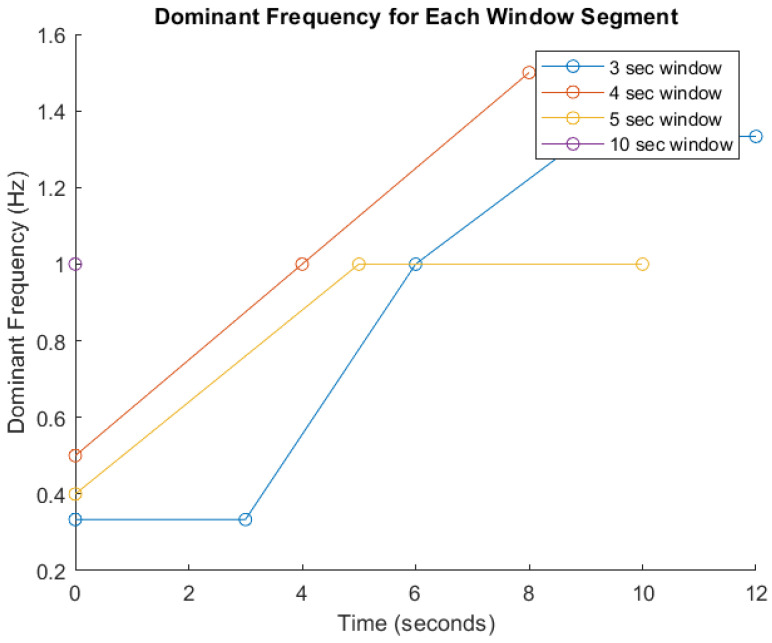
Dominant frequency analysis across segmented time windows. This figure illustrates the variation in dominant frequency over time for different signal segmentation window sizes (3, 4, 5, and 10 s) used in prehabilitation movement analysis. The x-axis represents time (in seconds), while the y-axis shows the dominant frequency (in Hz). Each colored line corresponds to a specific window duration: blue (3 s), red (4 s), yellow (5 s), and purple (10 s).

FFT-Based Signal Analysis: FFT is a well established method for analyzing time-series data, particularly for identifying frequency components in motion signals as indicated in many studies [20,21,46]. These features are critical for distinguishing between different types and intensities of physical activity [20]. Given the heterogeneity among participants in terms of age, body mass index (BMI), fitness level, height, underlying health conditions, and movement cadence, significant inter-individual variability in signal characteristics is anticipated. Therefore, amplitude and frequency parameters will serve as primary indicators for activity recognition, as they are foundational to most classification algorithms. To demonstrate the impact of segmentation window size on signal analysis, Figure 5 presents a comparative visualization of dominant frequency trends across different window durations (3, 4, 5, and 10 s). The graph shows how shorter windows (e.g., 3 s) yield faster responsiveness but result in broader and less distinct frequency spectra, potentially compromising classification accuracy. Conversely, longer windows (e.g., 5 and 10 s) capture more complete motion cycles and produce sharper frequency peaks, enhancing feature reliability but introducing latency. The 4 s window demonstrates a balanced trade-off between responsiveness and precision [20,21]. These trends, derived from preliminary simulations, underscore the limitations of fixed window segmentation and support the need for an adaptive strategy within the Digital Twin framework. By dynamically adjusting window sizes based on real-time signal characteristics, the system can optimize both responsiveness and critical accuracy for personalized pre-habilitation monitoring.

ML/AI-Based Data Interpretation: In parallel, ML/AI algorithms are applied to the same dataset to enhance pattern recognition and enable adaptive learning. Techniques such as supervised classification (e.g., decision trees, support vector machines) and deep learning models (e.g., convolutional neural networks, LSTM) are used to classify activity types and predict performance trends. These models are trained on labeled datasets and continuously updated using real-time sensor inputs, allowing for personalized feedback and dynamic system adaptation.

While FFT provides a robust foundation for frequency-based analysis, it is limited in its ability to capture complex, non-linear patterns and contextual variations. By integrating FFT features into ML/AI models, the system benefits from both spectral precision and intelligent interpretation. For example, FFT-derived features can be used as input vectors for ML classifiers, improving accuracy in activity recognition and anomaly detection. This hybrid approach of FFT, ML/AI, or FFT combined with ML / AI will demonstrate superior performance in terms of sensitivity, adaptability, and real-time responsiveness, making it well suited for personalized prehabilitation monitoring.

**Figure 6:**
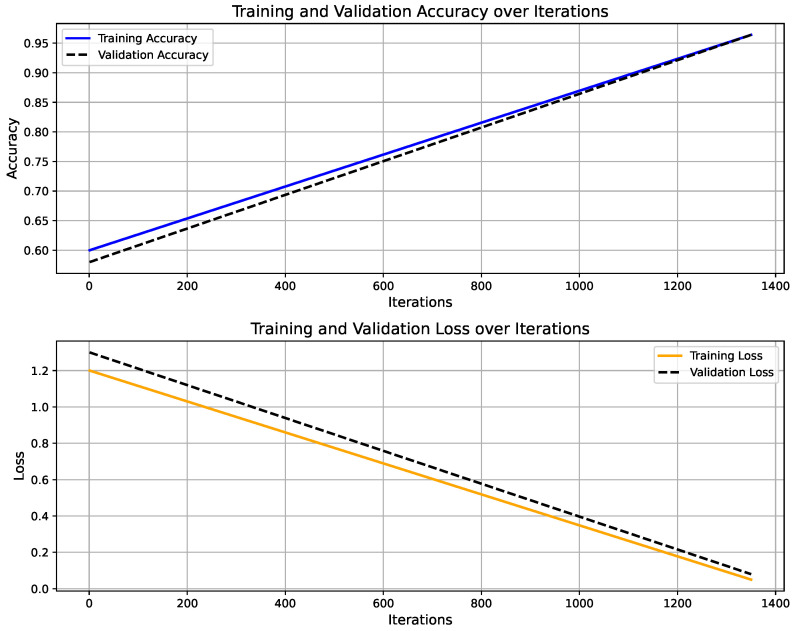
Training and validation performance of LSTM-based activity recognition model.

**Figure 7:**
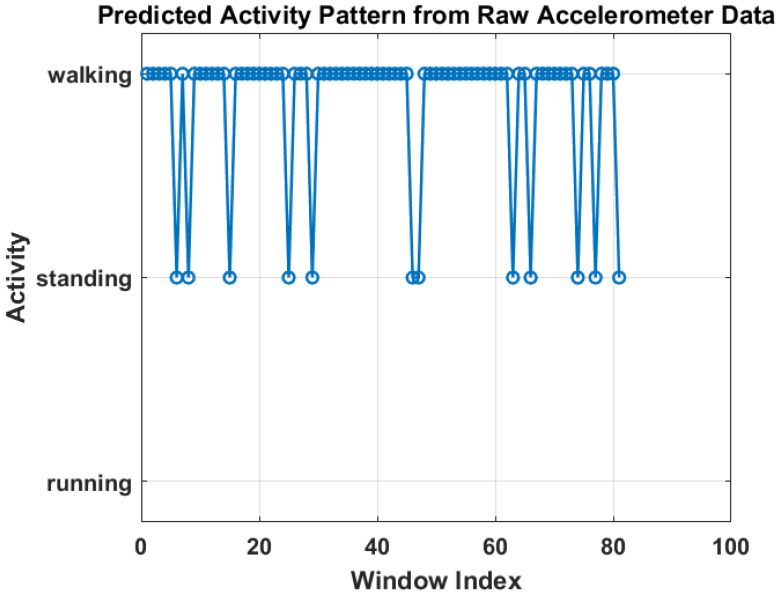
Predicted activity pattern from raw accelerometer data using low-weight AI model.

Figure 6 shows the training progress of the LSTM-based activity recognition model. The top graph illustrates the accuracy progression over 1350 iterations, with training accuracy (blue) and validation accuracy (black) both converging toward 100%, indicating strong model generalization. The upper panel presents the model’s accuracy curve for both training and validation datasets, achieving a final validation accuracy of 96.4% after 30 epochs using a constant learning rate of 0.001. Training was conducted on a single CPU over 1 min and 27 s. The lower panel depicts the corresponding loss convergence, where both training and validation losses decrease steadily, reflecting stable learning without overfitting. The overall trend confirms that the model successfully learns discriminative temporal patterns in sensor data, enabling reliable real-time activity prediction within the IoT–Digital Twin framework. On the other hand, Figure 7 illustrates the predicted activity pattern derived from raw accelerometer data using the AI model, where each window index represents a segmented data interval used for classification. The figure shows the alternating predictions between “walking” and “standing,” indicating that the model effectively distinguishes distinct motion states based on temporal sensor features. The consistency of detected activity windows demonstrates stable recognition performance and clear class separation across consecutive time segments.

These results represent preliminary testing of the proposed data processing framework. While the current analysis demonstrates the effectiveness of FFT-based signal processing and ML/AI techniques for activity recognition and system responsiveness, further research work will include the complete comparative analysis of algorithms required to validate the approach across a broader population. Future work will involve large-scale data acquisition from diverse user groups to assess the generalizability of the algorithms and enhance model personalization. This will support the development of more adaptive and clinically relevant Digital Twin models capable of delivering individualized prehabilitation interventions with higher precision.

## 6. Conceptual Progress Towards Digital Twin Implementation

As part of the conceptual framework, the integration of the Digital Twin component is envisioned to simulate patient-specific movement profiles and enable dynamic, personalized interventions. To support the rationale behind this framework, the paper presents a preliminary simulation illustrating the impact of varying signal segmentation windows on movement recognition accuracy.

**Figure 8:**
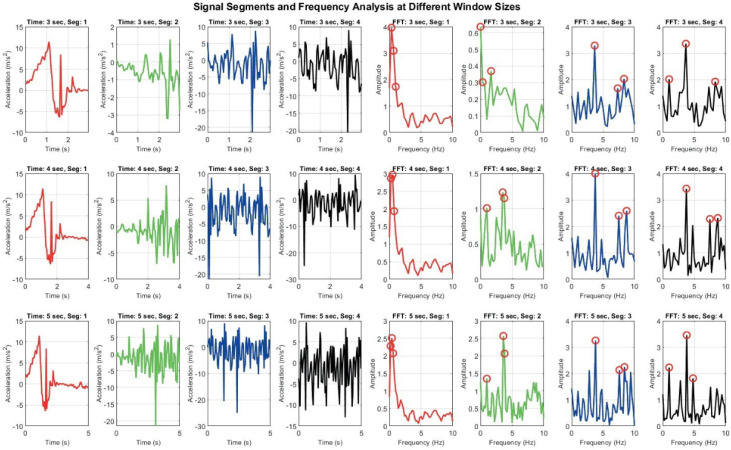
Signal Segmentation and frequency analysis at different window sizes: grid of plots showing time domain signal segments and their corresponding frequency domain representations using FFT across varying window sizes. Each row represents different time window (e.g., 3 s, 4 s, 5 s), while columns display segmented signals and their FFT outputs.

Figure 8 demonstrates how different window sizes (3, 4, and 5 s) affect both time domain signals and their frequency domain representations. A 3 s window offers faster responsiveness but results in broader, less distinct frequency spectra, which may hinder classification accuracy. In contrast, a 5 s window captures more complete motion cycles and yields sharper frequency peaks, enhancing feature reliability but introducing latency. The 4 s window represents a compromise between these extremes [20,21].

These observations, derived from a small-scale test sample, highlight the limitations of fixed window segmentation, particularly in diverse patient populations with varying movement patterns. They reinforce the need for an adaptive strategy in which the Digital Twin dynamically adjusts segment lengths based on real-time signal characteristics. Such adaptability is essential for improving recognition accuracy and responsiveness in personalized prehabilitation contexts. More broadly, these findings emphasize the importance of adaptability and automated intervention in activity recognition and real-time monitoring systems. While window size adjustment is one aspect, true adaptability encompasses multiple dimensions, including dynamic signal segmentation, personalized model tuning, and context-aware processing. Human motion is inherently variable, influenced by factors such as age, fitness level, injury status, and environmental context. Consequently, rigid, one-size-fits-all approaches may fail to capture the nuances required for accurate classification and timely intervention. An adaptable system should be capable of real-time decision-making, adjusting parameters such as sampling rate, feature extraction methods, and classification thresholds based on incoming data. This flexibility is particularly critical in Digital Twin applications for prehabilitation, where responsiveness and personalization are key. By continuously learning from sensor input and refining its models, such a system can provide more accurate feedback, detect subtle changes in physical performance, and support tailored prehabilitation strategies.

Ultimately, adaptability and dynamic management are not merely technical enhancements as they are foundational requirements for developing robust, scalable, and clinically relevant activity recognition systems. Given the significant variability in human movement across individuals and contexts, systems must be capable of adjusting in real time to maintain both accuracy and responsiveness.

**Figure 9:**
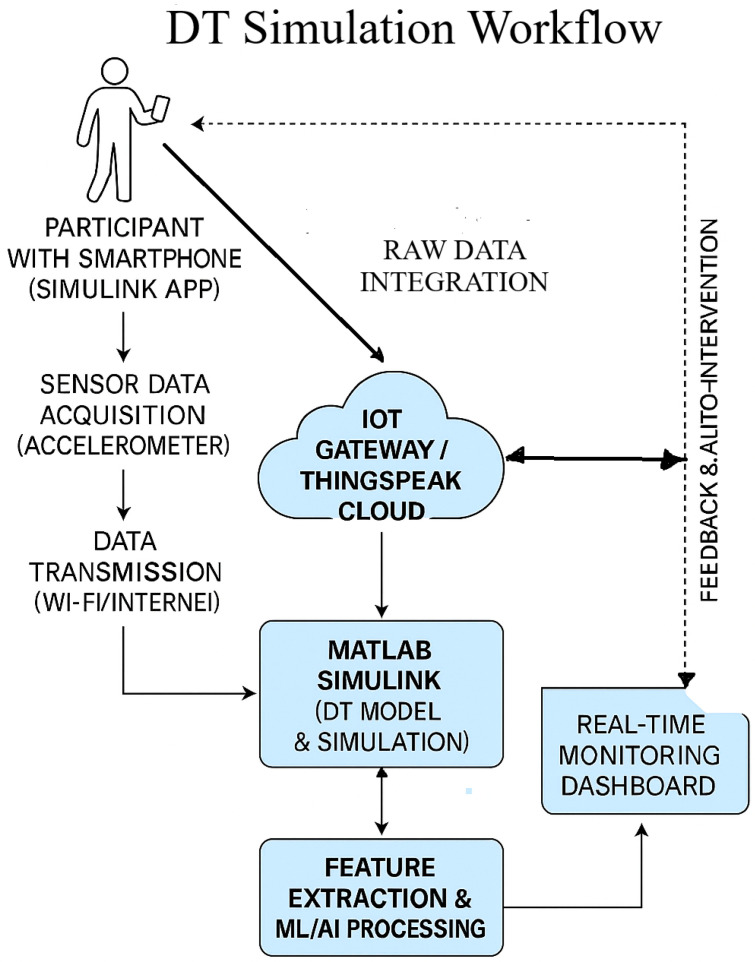
Digital Twin simulation workflow integrating mobile sensing, cloud computing, and real-time analytics for adaptive prehabilitation.

To facilitate real-time sensor data integration within the proposed DT framework, a structured and modular approach was adopted, employing the Simulink Mobile Sensor App in conjunction with the ThingSpeak cloud platform. A schematic overview of the Digital Twin simulation workflow is presented in Figure 9. In this configuration, participants carry smartphones equipped with the Simulink Mobile Sensor app to collect real-time accelerometer data, which are transmitted via Wi-Fi or mobile networks to an IoT gateway or the ThingSpeak cloud platform for ingestion and visualization. The acquired data are subsequently processed in MATLAB Simulink to construct and simulate the DT model, effectively transforming the smartphone into a wearable sensing device. Continuous and reliable data streaming between Simulink, ThingSpeak, and the edge gateway validates robust sensor–cloud integration.

ThingSpeak operates as the central cloud environment for data storage, visualization, and MATLAB-based analytics. The DT model follows up with the system in real time, providing a foundation for intelligent system adaptation. Planned enhancements include the integration of feature extraction methods and ML/AI algorithms to enable personalized feedback and automated interventions. These models will be trained on individual-specific movement patterns to support adaptive prehabilitation strategies for pre-operative abdominal patients. The subsequent phases of this research will focus on refining activity recognition models, enhancing system responsiveness, and incorporating adaptive algorithms capable of real-time learning and optimization of parameters.

This comprehensive integration not only enhances the system’s capability for continuous monitoring and adaptive feedback but also establishes a scalable and flexible architecture for future healthcare applications. By aligning real-world physiological data with virtual models, the proposed system enables a dynamic and personalized approach to prehabilitation. This foundational work sets the stage for subsequent phases of the study, where the DT framework will be further refined to support follow up of IoT system, automated decision-making, and individualized intervention strategies for pre-operative patients.

## 7. Conclusions

This paper presents a comprehensive review of the integration of Digital Twin, IoT, and ML/AI technologies in human movement monitoring systems, with a focus on prehabilitation for pre-operative abdominal patients. By synthesizing the current literature and technological advancements, the study identifies key functional components, challenges, and opportunities in designing intelligent, personalized movement recognition models. A use case of an IoT-based prehabilitation system is discussed, highlighting implementation aspects, limitations, and future directions. The findings from this study underscore the superior potential of ML/AI techniques over traditional frequency domain methods for accurate movement recognition, particularly in assessing movement type and intensity. A conceptual framework is proposed to demonstrate the synergy of DT, IoT, and ML/AI, addressing limitations of conventional IoT-based prehabilitation programs by enabling real-time monitoring, adaptive feedback, and personalized intervention planning. While the framework is primarily conceptual and literature-driven, it lays the groundwork for future empirical validation and implementation. The integration of DT technology not only allows for follow up of the IoT system but also is expected to enhance adaptability and responsiveness by simulating patient-specific movement patterns and enabling dynamic interventions. This is particularly beneficial for elderly patients with limited access to in-person care.

Future work will focus on data acquisition on pre-operative abdominal patients populations, prototype development for real-time sensor–DT integration, and refinement of ML algorithms for personalized analytics. These efforts aim to transition the framework from theory to practice, improving movement recognition, personalization, and adaptability in IoT-based prehabilitation systems. Ultimately, this approach has the potential to reduce healthcare costs and improve prehabilitation functional outcomes for pre-operative patients in mixed-mode settings.

## Figures and Tables

**Table 1:** Summary of reviewed studies on prehabilitation programs.

Review Study	Population	Duration	Type of Exercises	Key Functional Outcomes
Supervised Prehabilitation				
[3,4]	Colorectal cancer patients	4–6 weeks	Aerobic and resistance training, flexibility exercise	20% increase in 6MWT; 35% reduction in post-operative complications
[7]	62 candidates (patients)	17.5 sessions (2 sessions/week)	High- and moderate-intensity	aerobic fitness improvement, strength, and quality of life; lower risk of surgical failure in exercise group (5% vs. 21%)
[11]	Review study	—	Low-, medium-, and high-intensity exercises	Significant improvements in physical activity scores and walking test results, indicating better physical readiness for surgery
[12]	14 patients	3 sessions/week for 3 weeks	Low-volume HIIT program	13% increase in VO_2_ peak; strong correlation between walking distance and VO_2_ peak ($R^{2}=0.52$, *p* < 0.001)
Unsupervised/Technology-Based Prehabilitation				
[13]	172 participants	4–8 weeks	Aerobics, resistance, and respiratory exercises; recommendations of home exercises	Improved physical and psychological readiness for surgery; potentially improving post-operative outcomes
[5]	204 randomized patients (out of 543 assessed)	5 weeks	Home-based walking	No significant improvement in functional recovery or other outcomes compared to standard care
[8]	80 patients scheduled for colorectal cancer resection	—	—	20 m improvement in 6MWT; post-operative complications assessed
[14,15,16,17]	Abdominal cancer patients	4–6 weeks	Low to high cardiorespiratory fitness testing using treadmill	Adherence and outcomes of prehabilitation assessed
[20,22,38]	Abdominal cancer patients	4–6 weeks	Low, medium, high aerobic exercises	Remote monitoring and feedback alert system applied

**Table 2:** Key elements and boundaries of prehabilitation programs [22].

Sl.No	Prehabilitation Elements	Boundaries	Remarks
1	Prehabilitation Program Duration	4–6 weeks/4–8 weeks	Patient’s status and surgical schedules
2	Number of Sessions Per Week	2 or more	Can participate as per the guidance of health supervisor
3	Threshold Duration	150 min of moderate duration or equivalent	75 min of vigorous intensity or a combination of vigorous and moderate exercise
4	Minimum Duration of Each Session	10 min or more at moderate intensity	As per patients’ needs
5	Initial Assessment	6MWT, cardiopulmonary exercise testing, 10-m shuttle walk test	Dependent upon clinical resources and expertise
6	Exercises Involved	Walking, cycling, treadmill and land-based running, cross-trainer, staircase ascending and descending, rowing, step-up, leg press	Can be altered according to need
7	Location	Healthcare center, clinic, gym, indoor, sports club or park	Availability of resources
8	Performance Measurement	Credit Point Calculation	Not standardized; conceptual analysis of performance based on credit point calculation

**Table 3:** Studies on sensor-based activity recognition and application and recognition outcomes.

Study	Technology Used	Application	Performance/Technique
[20,23]	Accelerometer, IMU, IoT-enabled devices	Lower body and transitional activities	FFT, 4 s window; 78% accuracy
[47]	Smart mobile sensors (accelerometer, gyroscope, magnetometer)	Walking, brisk walking	Deep learning model; 96.5% accuracy
[45]	Smartphone embedded sensors with classifier	Daily activities (standing, sitting, lying, stairs, walking)	FFT and ML with 3 s window; PCA—96.11%, Frequency domain—92.10%
[49]	Smartphone with ML and DL	Static and dynamic activities	model survey
[51]	Waist-mounted inertial sensor (accelerometer and gyroscope)	Real-time activity recognition	Adaptive window; 96.4% accuracy

**Table 4:** Summary of Digital Twin applications integrating IoT and ML/AI technologies in healthcare and prehabilitation.

Study	Application Domain	DT Integration Type	Technologies Used	Key Outcomes
[24,25,26,34]	Engineering and Healthcare	Conceptual Overview	DT architecture, communication protocols	Defined DT components and cross-domain applications
[29]	Sports and Rehabilitation	Behavioral and Physical Modeling	IoT sensors, user input apps	Personalized recommendations and predictive modeling
[32]	Elderly Care	Cloud DT	Wearable, IoT, Cloud analytics	Real-time monitoring, tracking, and predictive interventions
[31]	Remote Kinesiotherapy	Virtual Supervision	DT simulation	Remote control and risk prevention in therapy
[30]	Healthcare	Wearable DT/Edge DT	Real-time monitoring	
[33]	Healthcare, ECG Monitoring	AI-empowered DT	VGG, LSTM, AI, DT	Real-time ECG anomaly detection and adaptive feedback

## Data Availability

The original contributions presented in this study are included in the article. Further inquiries can be directed to the corresponding author.
